# Teeth with Dead Pulps, without Fistule, and the Filling of Roots

**Published:** 1888-10

**Authors:** J. Morgan Howe

**Affiliations:** New York.


ARTICLE V.

### TEETH WITH DEAD PULPS, WITHOUT FISTULE, AND THE FILLING OF ROOTS.

DR. J. MORGAN HOWE, NEW YORK.

In the consideration of the subject I shall not weary you by attempting to be exhaustive, but will touch on some salient points concerning which there is perhaps the greatest disparity of opinion. Leaving our special consideration of the teeth whose pulps have been intentionally devitalized by the dentist, we have those whose pulps have been dead for an indefinite period, presenting various phases and phenomena which ought to be interesting, and are certainly troublesome and disconcerting to the practitioner. One prerequisite to the successful treatment of all teeth whose *pulps are dead*, and one which seems to be too little appreciated, is to obtain as direct access to the apical end of the pulp canal as is practicable by drilling or cutting away any portion of the tooth that obstructs, or makes such access more difficult. No part of the tooth need be recklessly sacrificed; but no hesitation should be felt in cutting away sufficient to permit the most thorough removal possible of all disorganized matter from the canals, and the most perfect possible filling of the canals afterward. Of what benefit can it be to spare a portion of enamel or dentine which in any degree prevents such necessary procedures, and thereby increases the probabilities of future irritation of peridental tissues, and may cause the ultimate loss of the tooth? The sacrifice of a large part of the crown of any tooth, if necessary to attain the end, would be preferably to jeopardizing the health of the root. These remarks, however, are not intended as an indorsement of the practice of using engine burs in root canals any further than can be done with certainty that they are following the canal; and this would restrict them to a limited use, in merely enlarging or making more accessible the entrance into them. Educated fingers are often unreliable when the reaming tool is directly in their grasp, but if the engine hand piece is around the rapidly revolving tool, they can tell almost absolutely nothing as to where the point is going. The *greatest* care is desirable in all efforts to enlarge canals to avoid passing a drill through the root of a tooth at any other point than at its apex, and even this is undesirable unless *required* for drainage.

Dead pulps may be opened to external influences, or closed in by fillings, or by unbroken walls of dentine. In either, the teeth may be perfectly quiescent and comfortable, or may be the location of any grade of pericementitis. Disorganisation of the pulp tissue takes place after loss of vitality, but *putrefaction* cannot occur with access of micro-organisms which promote this process. This condition being fulfilled in an exposed dead pulp, it may always be assumed that it is putrescent. Free exit of this mephitic matter into the mouth, with much more difficulty of its escape through the apical foramen may, and often does result in the tooth remaining free from perceptable pericemental irritation for an indefinite period, but when these mechanical conditions are relatively changed, the tissues of the socket suffer from contact of putrefactive organisms of the poisonous results of their existence, and inflammation comes as an acute of chronic process. Acute pericementitis under these conditions may generally be terminated in its earlier stages by removing all debris from the root canals, so as to reopen this obstructed vent. When it has passed the stage in which this procedure gives relief, non-irritant antiseptics, as iodoform in solution, or creosote, or an anodyne, as wine of opium, applied in the root canal may be effective, and counter-irritation applied to the gum is sometimes helpful, but these expediments are perhaps as likelv to fail as to succeed ; active constitutional treatment if intelligently used will generally be much more effective. The recognition that has been given to the efficacy of systematic treatment, in the new " American System of Dentistry," is, I think, a gratifying evidence of progress. Chronic pericementitis frequently results in abscess by the time the case comes under treatment, and a blind alveolar abscess is a condition that—I feel warrented by experience in saying—should be treated with *distinguished* consideration. Haste in permanently closing the aperture through which any abscess has had vent or drainage is always attended with risk of unfavorable consequences; and in the case of alveolar abscess, though immediate symptoms of irritation may not result, we are not on that account warranted in proceeding to fill the root till we are reasonably certain the abscess is destroyed, and the process of repair has begun. The cleansing of pyogenic tissues and the application directly to them—if that were possible in these cases—of antiseptic or other medicaments, would be no assurance that suppuration will not continue, and would not therefore justify proceeding as if it had actually been arrested, and the process of repair begun.

So much patience is often required in dealing with such abscesses, and so great are the difficulties of evacuating the pus and applying successful treatment to the pus cavity through the foramen of the root, that wearied with the monotony of unfavorable condition, we are prepared on such occasions if ever, to resort to the use of some new remedy, which some one has somewhere recommended as the best of all. I have in this way discovered in what irritable mood the tissues sometimes are, that constitute the walls of a blind abscess. After using tereben several times for deodorizing or disinfecting root canals—which it does—an obstinate case of chronic, blind abscess, in connection with a lower bicuspid, induced me to apply this drug in the canal of the tooth. Within three hours pain was severe, and acute inflammation resulted, continuing thirty-six hours before I succeeded in arresting it. A similar result, much less severe in degree, has several times resulted from my use of peroxide of hydrogen in efforts to cleanse blind abscess, and eucalyptol recently roused the inflammatory fires, in an abscess of this kind, with such manifestations that I was persuaded to remove the tooth, whose chances of permanent usefulness I had previously considered rather poor. These may be exceptional instances of the action of these drugs, for they have been recommended for the condition; they serve to illustrate my statement, however, that a blind abscess presents an irritable condition of the tissues, and had better be treated with medicaments incapable of producing such results as I have described. I have known carbolic acid to produce irritation in these, but not so severe and persistant as the remedies before mentioned. The treatment that has been most uniformly successful in my hands has been to remove all debris possible from canals, and if no purulent discharge can be perceived, dress loosely in the canal with etherial solution of iodoform, covering with cotton, in which very little sandarac or other gum varnish is absorbed. If this produces no discomfort from pressure of purulent matter or mephitic gases in forty-eight hours or more, the same dressing may generally be sealed in with gutta-percha, and the case conducted thence by one or more repetitions to a speedily successful termination, with root-filling; but, if on cleansing the canal a discharge can be observed coming through the apical foramen, I would apply no medicament, but stop the cavity loosely with cotton, to secure drainage of the abscess and exclude food from the cavity, and later follow with the treatment before described, but being more careful to allow some discharge by applying the dressing loosely.

Ordinary, blind abscesses will generally be cured by such treatment without unusual delay, but when suppuration has been long continued and the apex of the root is denuded of pericementum by the destructive process, the chances of cure are much less, because the end of the root acts like a foreign body in the tissues; in many cases it is eroded by the purulent accumulations, or has granular calculus deposited on it, and in most cases, whether these latter conditions are present or not, as an irritant which tends to promote continued suppuration, so that the most certain way of effecting a cure is to amputate the portion of the root thus affected. I have always heretofore accomplished this with a bur, with the tooth *in situ*.

When a dead pulp is sealed in from external influences, the tooth may remain for years without giving perceptible offence to the surrounding tissues, and unnoticed by its owner. During this time the pulp has been disorganized; but, as before remarked, putrefaction has not taken place unless the micro-organisms peculiar to this process have had access to the pulp canal. I infer that during the period in which the nutrition of the percimental tissues is not affected, *bacteria* are not present, but that irritation, inflammatory changes aud suppuration are easily accounted for by the theory that organisms or spores have found entrance through the apical foramen to a condition so favorable to their proliferation, and that the immensely multiplied organisms themselves, or the poisonous products of their existence, become the means of injury to the peridental tissues.

When adead pulp, sealed and without external change of condition, becomes a cause of irritation, whether immediately after devitalization, or indefinitely later—following a period of quiescence—I infer that bacteria or spores have been brought to the apical foramen, in the blood or otherwise, and that to their agency is due the changes which have caused inflammation or suppuration. It is noticeable clinically that enervated conditions, or systematic debility favor the access of such inflammations, which frequently occur after long periods of immunity, notwithstanding the presence meanwhile of the dead pulp. But perceptible irritation is not always in these cases the beginning of injury; we notice this peculiarity, that pus is often already formed when the patient applies for relief, and that the common and best procedure,—drilling a vent,—yields a purulent discharge, though the inflammatory manifestations may have been present only a few hours. With these, and those in which the dead pulp is exposed, chronic abscesses with considerable destruction of tissue, may be formed at the apex of the root, without a perceptible inflammatory process, and the real condition perhaps be discovered only when acute symptoms lead to diagnosis and treatment; as if bacterial proliferation in such favorable condition were capable of producing suppuration without the phenomena of inflammation preceding, but coming after as a result of the extension or acceleration of the former process.

Since 1882 I have practiced the method suggested to me by Dr. Bodecker—since published by him—of making an opening, just giving access to the pulp cavity, and without the least effort to remove disorganized matter, introducing quickly some solution of iodoform in ether, or ether in alcohol, on a loose tent of cotton, and sealing immediately with gutta-percha. After three or more days the root canals are cleansed and dressed with the same solution, and again sealed, and a few days after this second application the filling of the roots may be accomplished. By following this method I have found such exemption from irritation of peridental tissues that I may say there has not been a single failure; the few instances in which some pain followed opening the teeth resulted, in my opinion, from my undue confidence leading me to remove some of the pulp debris before making the antiseptic application. In these pain subsided in an hour or two after it began, and without treatment, or any subsequent unfavorable results. These latter incidents seem to me to be strong additional evidence of the power of iodoform to prevent the progress of putrefaction and the pericemental irritation resulting, which is notably so difficult to arrest; for before the use of iodoform in these cases I had almost never known this inflammation to yield to treatment satisfactorily; but with such results invaribly following this preventive method, I have for a long time regarded the opening of such teeth as an operation attended with no risk. I do not hesitate to open any such tooth that needs treatment. I have already suggested an explanation, that in a dead pulp causing no irritation putrefaction has not occured; but admission of air and these life germs cause it to begin immediately, and the bacteria or poisonous products of their life produce the irritation; and as they are also the organisms of suppuration, the latter is difficult to prevent when inflammation is commenced under such circumstances.

Iodoform fulfils the requirements, and does not produce any irritation in concentrated solution, which cannot be said of other substances that approach it in antiseptic power. If disinfection or changing of a substance that is poisonous to an innocuous condition, were what we require, how shall we account for the fact that poison has been in the root for years and has not irritated the peridental tissues, but will do so within a few hours after admission of the atmosphere unless measures are employed to prevent it? Or if sulphuretted hydrogen is the cause of the disturbed nutrition we have to fear, how can we account for the fact that the vent which would be so necessary for its escape is shown to be unnecessary?—*N. Y. Trans.*

